# The "Plague of Women"

**Published:** 1900-05-19

**Authors:** 


### THE "PLAGUE OF WOMEN."

Speaking at the last meeting of the Royal Medical and Chirurgical Society, and referring to the "plague of women," of which Mr. Treves had complained as infesting the hospitals in South Africa, Surgeon General Jameson announced that already Mr. Treves' remarks had borne fruit, for he was informed that the engineers had encircled the hospital containing cases of enteric fever with barbed wire!

